# Synthesis and Characterization of Multilayered CrAlN/Al_2_O_3_ Tandem Coating Using HiPIMS for Solar Selective Applications at High Temperature

**DOI:** 10.1021/acsaem.3c02310

**Published:** 2023-12-29

**Authors:** Miriam Sanchez-Perez, Teresa Cristina Rojas, Daniel F. Reyes, F. Javier Ferrer, Meryem Farchado, Angel Morales, Ramon Escobar-Galindo, Juan Carlos Sanchez-Lopez

**Affiliations:** † Instituto de Ciencia de Materiales de Sevilla (CSIC-Univ. Sevilla), Avda. Américo Vespucio 49, E-41092 Sevilla, Spain; ‡ University Research Institute on Electron Microscopy and Materials (IMEYMAT), Universidad de Cádiz, E-11510 Puerto Real (Cádiz), Spain; § Centro Nacional de Aceleradores (Univ. Sevilla, CSIC, and Junta de Andalucía), Avda. Tomás A. Edison 7, E-41092 Sevilla, Spain; ∥ Departamento de Física Atómica, Molecular y Nuclear, Universidad de Sevilla, Aptdo 1065, E-41012 Sevilla, Spain; ⊥ CIEMAT-PSA, Materials for Concentrating Solar Thermal Technologies Unit, Avenida Complutense 40, E-28040 Madrid, Spain; # Departamento de Física Aplicada I, Escuela Politécnica Superior, Universidad de Sevilla, Virgen de África 7, E-41011 Sevilla, Spain

**Keywords:** selective absorption, optical properties, emissivity, thermal stability, bias, sputtering

## Abstract

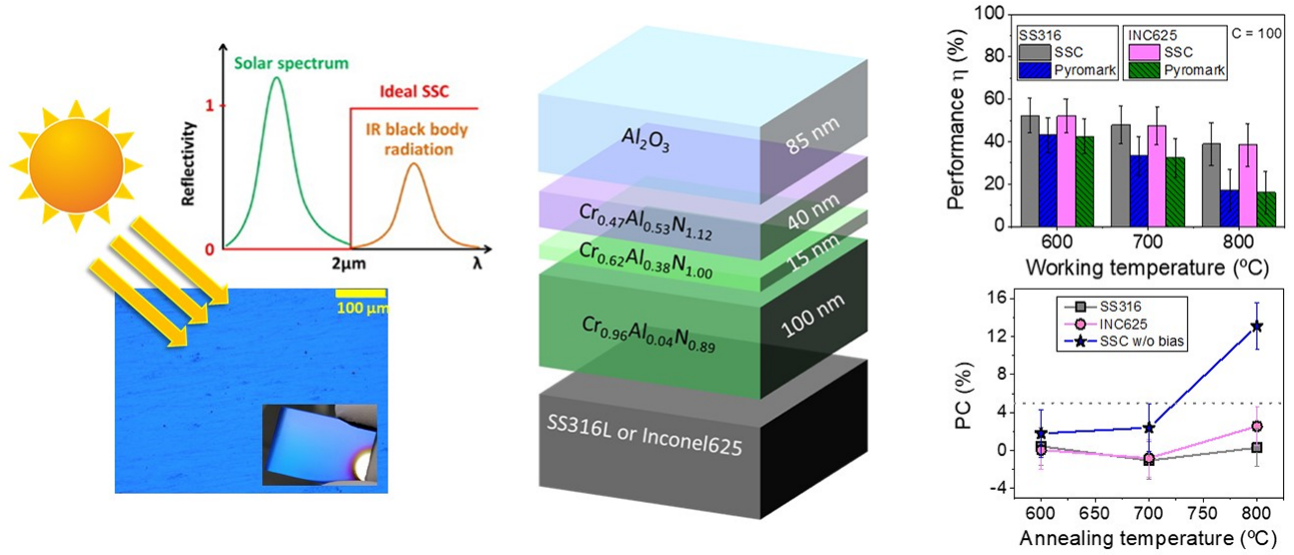

The effect of applying a negative bias during deposition of a previously designed multilayer solar selective absorber coating was studied on two types of substrates (316L stainless steel and Inconel 625). The solar selective coating is composed of different chromium aluminum nitride layers deposited using a combination of radiofrequency (RF), direct current (DC), and high-power impulse magnetron sputtering (HiPIMS) technologies. The chemical composition is varied to generate an infrared reflective/absorber layer (with low Al addition and N vacancies) and two CrAlN intermediate layers with medium and high aluminum content (Al/Cr = 0.6 and 1.2). A top aluminum oxide layer (Al_2_O_3_) is deposited as an antireflective layer. In this work, a simultaneous DC-pulsed bias (−100 V, 250 kHz) was applied to the substrates in order to increase the film density. The optical performance, thermal stability, and oxidation resistance was evaluated and compared with the performance obtained with similar unbiased coating and a commercial Pyromark paint reference at 600, 700, and 800 °C. The coating remained stable after 200 h of annealing at 600 °C, with solar absorptance (α) values of 93% and 92% for samples deposited on stainless steel and Inconel, respectively, and a thermal emittance ε_25°C_ of 18%. The introduction of additional ion bombardment during film growth through bias assistance resulted in increased durability, thermal stability, and working temperature limits compared with unbiased coatings. The solar-to-mechanical energy conversion efficiency at 800 °C was found to be up to 2 times higher than Pyromark at *C* = 100 and comparable at *C* = 1000.

## 1 Introduction

Despite climate change warnings, carbon-based fossil sources continue to be the dominant supplier of the world’s total primary energy supply (TPES). In the period from 1971 to 2018, renewable energy sources (hydro, wind, and solar) only contributed 4.5% of the world’s TPES.^1^ Hence, the development of new technologies for large-scale production of electricity using renewable sources has to be boosted. This holds specifically for solar energy, including photovoltaics (PV) and concentration solar power (CSP, also known as solar thermal electricity). CSP technology concentrates the sun radiation flux using a heliostat field that is received by a heat collector (solar receiver). The solar receiver converts this radiation into heat using a heat-transfer fluid (HTF), as saturated steam or molten salts, and finally, this heat generates electricity using a turbine and a generator. CSP allows the storage of heat, enabling solar thermal electricity to be dispatched on demand day and night.^2−4^ The main CSP configurations are parabolic trough collector (PTC) and central receiver solar power plants. PTC operates commercially at a maximum temperature of 400 °C with the solar absorber tube encapsulated in a vacuum. The present upper limit temperature of the central receiver solar power plants is 565 °C, which falls below the ideal temperature range. Hence, the development of new materials operating in air at higher temperatures (*T* > 800 °C) is currently an intense research field to enhance the global efficiency of the plants.

In this regard, the use of a solar selective coating (SSC) deposited on the solar receivers significantly improves the performance of the CSP system.^5−11^ A desirable SSC should exhibit excellent solar absorptance (α ≥ 95%) and minimal thermal emissivity (ε ≤ 20%) and maintain thermal stability even under high operating temperatures. Currently, SSCs deposited by physical vapor deposition (PVD) techniques are commercially used in PTC plants. However, the harsher operation conditions of central receiver plants hinder, so far, the use of SSCs in this technology, and silicone-based paints like Pyromark-2500 are utilized as solar absorbers. However, Pyromark-2500 exhibits low solar selectivity attributed to its inherently high thermal emittance, and it also experiences rapid degradation when operated at temperatures exceeding 700 °C.^12^ As a result, frequent maintenance and repair, typically every two years, are necessary to ensure its optimal performance. Hence, in recent years, there has been a significant research effort focused on creating novel SSCs that possess exceptional temperature stability in oxidizing atmospheres.^13−17^

In particular, transition-metal-based nitrides, oxynitrides and oxides, have been extensively studied as SSC candidates due to their exceptional resistance to oxidation, chemical and corrosion properties, and tunable optical properties. These coatings can be combined into multilayer structures to optimize their solar performance exhibiting high absorptance (up to 98%) and reduced emittance (<15%). Some high-temperature solar absorber coatings that have already been reported are CrAlN/CrAlON/Al_2_O_3_, CrAlN/CrAlON/Si_3_N_4_,^18^ Ti/AlTiN/AlTiON/AlTiO,^19^ TiN/AlCrSiO/AlCrSiO,^20^ AlMoN(H)/AlMoN(L),^21^ Mo–Si_3_N_4_,^22^ Mo/ZrSiN/ZrSiON/SiO_2_,^23^ W/Ag/WN-AlN/AlN/SiO_2_,^24^ W/AlSi_*x*_N/AlSiO_*y*_N_*x*_/AlSiO_*x*_,^25^ W/CrAlSiN_*x*_/CrAlSiN_*x*_O_*y*_/SiAlO_*x*_,^26^ and W/WAlSiN/SiON/SiO_2_.^27^ In addition, a table presenting the solar selectivity of metal nitride/oxynitrides SSC coatings deposited by magnetron sputtering on various substrates can be found in ref (28). In previous works, Escobar-Galindo et al. studied the microstructure, element composition, chemical bonding, and optical properties of Al_*y*_Ti_1–*y*_(O_*x*_N_1–*x*_) multilayered coatings, both before and after single-stage (12 h) and thermal cycles in air.^29,30^ The findings revealed that the SSCs remained stable up to 650 °C during the single-stage tests. However, at 800 °C, a rutile-TiO_2_ film formed on the surface, causing the coating to degrade. Then, the samples underwent symmetric thermal cycles in air, involving heating and cooling ramps of 10 °C/min, with temperatures ranging between 300 and 600 °C, fulfilling the performance criterion of PC ≤ 5% for 300 cycles (total cycling time 900 h). More recently, we have developed a multilayered system based on chromium aluminum nitride layers whose Al content is increasing progressively from the bottom to the top and ended with an alumina layer for antireflective purposes.^31,32^ The high Al content of the top Cr_1–*x*_Al_*x*_N_*y*_ layer (Cr_0.47_Al_0.53_N_1.12_), along with the protective Al_2_O_3_ layer on the surface, makes these SSCs well-suited to provide oxidation resistance. Samples were proved to be stable after annealing up to 600 °C in air over 2 h, maintaining α ≈ 94% and ε_25°C_ < 15%. Utilizing high power impulse magnetron sputtering (HiPIMS) technology, film density and functionality can be enhanced through the application of short pulses, typically ranging from 50 to 500 μs, at low frequencies below 1 kHz. This enables the achievement of high peak current (>1 A/cm^2^), peak power densities (0.1–3 kW/cm^2^), elevated plasma densities (10^19^ m^–3^), and ionization rates exceeding 40%.^33^ The densification of the film microstructure thanks to the ion bombarding during plasma synthesis is expected to enhance the oxidation resistance and thermal stability by retarding the ion interdiffusion and oxygen inward penetration. In this paper, the SSC multilayered architecture previously developed is also grown with HiPIMS but assisted with an additional ion bombardment provided by negative biasing of the substrates. The dependence of the optical performance and thermal resistance is evaluated comparatively at 600, 700, and 800 °C over 2 h with the stack grown without bias on two different substrates (stainless steel 316L and Inconel 625) and Pyromark as a reference. Longer annealing times at 600 °C over 200 h were tried, confirming the enhancement of the thermal stability and higher solar-to-mechanical efficiencies than the commercial paint used, Pyromark.

## 2 Experimental Section

### 2.1 Multilayer Coating Preparation

A multilayered stack, CrAlN_1–*x*_/CrAl(Lo)N/CrAl(Hi)N/Al_2_O_3_, was grown via a combination of high-power impulse (HiPIMS), radiofrequency (RF-MS), and direct current magnetron sputtering (DC-MS) sources using chromium (99.95% purity) and aluminum (99.999% purity) targets of 2 in. diameter provided by Photon Export. Chromium was sputtered using a HiPIMS source (Solvix) at 300 W, a pulse of 40 μs, and 500 Hz of frequency. An aluminum target was connected to an RF source (Huttinger) at variable power (from 40 to 300 W) depending on the specific individual nitride layer (cf. Table 1). A DC-pulsed (DC-p) power operating at 250 kHz (88% of duty cycle) was employed for applying an average negative bias of 100 V during deposition of the nitride layers. An alumina top layer was placed as antireflective material, which was deposited by reactive sputtering deposition using the RF source at 250 W. Coatings were deposited on 316L stainless steel (hereafter SS316), Inconel 625 (hereafter INC625), and silicon (100) substrates, with a root-mean-square roughness (RMS) of 62, 42, and 6 nm, respectively. Table 2 shows the chemical composition of the metallic substrates provided by the companies. The base pressure of the vacuum chamber was 2 × 10^–4^ Pa, and the working pressure was set at 0.9 Pa. More details on the deposition procedure can be found elsewhere.^31,32^

**Table 1:** Nominal Chemical Compositions, Synthesis Conditions, and Film Thickness of Different Layers Constituting the Multilayered Solar Absorber Stack

code	nominal	Ar (sccm)	N_2_ (sccm)	O_2_ (sccm)	Cr (W)	Al (W)	bias (V)	thickness (nm)
C1	Cr_0.96_Al_0.04_N_0.89_	20	4		300	40	–100	100
C2	Cr_0.62_Al_0.38_N_1.00_	20	4		300	150	–100	15
C3	Cr_0.47_Al_0.53_N_1.12_	20	5		300	300	–100	40
Al–O	Al_2_O_3_	25		2.5		250	floating	85

**Table 2:** Chemical Composition of the Stainless Steel 316L and Inconel 625 Substrates Expressed in wt %

	SS316	INC625
C	0.08	0.10 max.
Mn	2	0.50 max.
Si	0.75	0.50 max.
Cr	16.00–18.00	20.0–23.0
Mo	2.00–3.00	8.0–10.0
Ni	10.00–14.00	58.0 min.
W	6.42	
Nb(+Ta)		3.15–4.15
V	1.95	
Co		1.0 max.
Al		0.40 max.
Ti		0.40 max.
Fe	Bal.	5.0 max.

**Figure 1:**
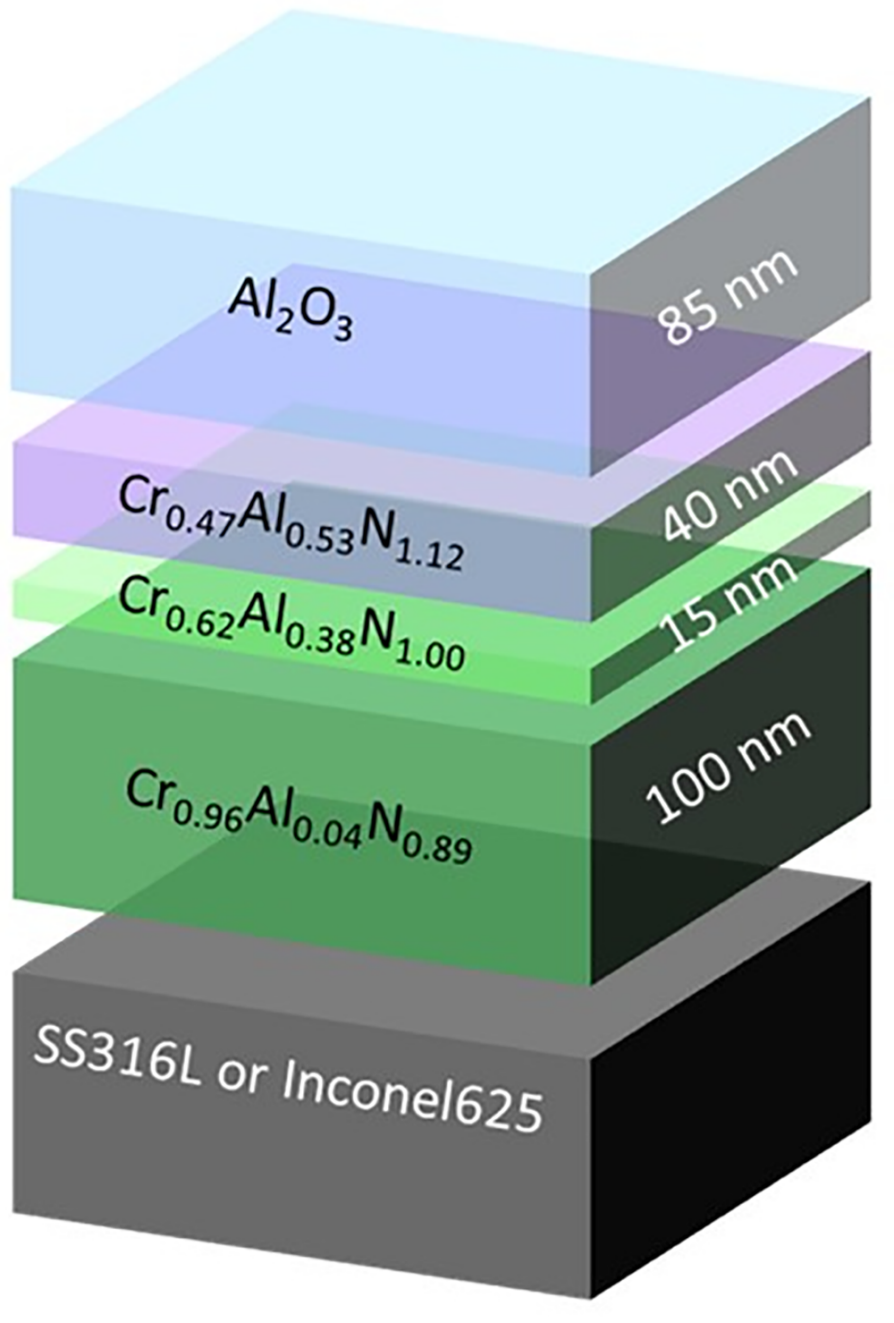
Schematic representation of the designed SSC multilayered stack.

A schematic representation of the multilayered stack including specific thicknesses is plotted in Figure 1. The given stoichiometries are nominal, and their thicknesses correspond to an optimized stack previously published by our group.^31,32^ This was replicated in this work by the simultaneous application of a negative bias on the sample holder while keeping a single batch process strategy. The synthesis conditions used to reproduce the nominal stoichiometries are detailed in Table 1.

### 2.2 Thermal Annealing Treatment

The coatings were subjected to short-term isothermal annealing treatments in a muffle furnace in air following the same procedure described in refs (31) and (32). Samples were heated for (i) 2 h up to 600, 700, and 800 °C and (ii) for 200 h at 600 °C. A heating rate of 5 °C/min was used for all thermal treatments. Pyromark coupons were used as a reference by painting both types of metallic substrates with this commercial material (estimated thickness 25 μm).

### 2.3 Microstructural and Compositional Characterization

Grazing incidence X-ray diffraction (GIXRD) measurements were performed at (1°) using Cu Kα radiation in an X’Pert Pro PANALYTICAL diffractometer to obtain diffraction patterns of the as-prepared and after-thermal-treated SSCs deposited on INC625 and SS316.

Cross-section scanning electron microscopy (X-SEM) was done by using a HITACHI-S4800 high-resolution field emission gun (FEG) microscope. X-SEM views allow the study of the thickness and morphology of SSCs deposited on silicon substrates.

A dual focused ion beam FIB (Dual-Beam Helios) was employed to fabricate cross-sectional lamellas of the SSCs deposited on the metallic substrates for the transmission electron microscopy (TEM) characterization. FEI Talos F200X and double aberration-corrected Titan Cubed3 Themis microscopes working at 200 kV were used in this work. High angle annular dark field scanning TEM (HAADF-STEM) images and nanoprobe X-ray energy dispersive (EDX) analysis were performed to study the microstructure and chemical composition of the SSCs. EDX maps were obtained by using ChemiSTEM Technology with four integrated Bruker SDD detectors and processed using Velox software.

A LabRAM Horiba Jobin Yvon spectrometer with a diode-pumped solid state laser (532 nm) at 5 mW and a CCD detector was used to obtain Raman spectra of the SSCs in the 200–1000 cm^–1^ range. An exposure time of 100 s and an aperture hole of 100 μm were employed in all measurements.^31,32^

Ion beam analysis (IBA) using Rutherford backscattering spectrometry (RBS) was performed at the “Centro Nacional de Aceleradores” (CNA) to obtain the chemical composition of the samples. Measurements were taken using α particle beams, at different incident energies, with a passivated implanted planar Si detector placed at a laboratory angle of 165°. RBS measurements at 1.0 and 2.0 MeV were used to determine the Cr and Al stoichiometry, while the N content of the samples was obtained using the higher sensitivity of the technique at an energy of 3.7 MeV, due to the broad resonance in the N(He,He)N cross section.^31,32^ IBA spectra were analyzed using SIMNRA software.^34^

### 2.4 Optical Characterization

A UV–vis–NIR Cary 5000 spectrometer with an integrating sphere of 100 mm of diameter was used to obtain the reflectance of the SSCs in the 250 nm to 2.5 μm range, while a PerkinElmer Frontier Fourier Transform Infrared (FTIR) spectrophotometer provided the reflectance in the mid-IR range (2.5–17 μm). These optical measurements allowed the calculation of the solar absorptance α and thermal emittance ε following the equations:^35,36^
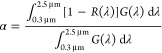
1
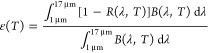
2where *R*(λ) stands for the spectral reflectance of the samples, *G*(λ) is the solar radiation power at AM1.5, and *B*(λ,*T*) represents the spectral blackbody emission at temperature *T*.^37^ The reflectance measurement uncertainty was estimated to be 0.5% and 1% for the UV–vis and FT-IR spectrophotometers, respectively.

The performance of the developed SSCs can be estimated by using the following parameters:

The solar performance, or solar-to-mechanical energy conversion efficiency (η), that is defined by the expression:^38^
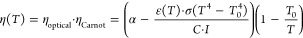
3where *C* defines the solar concentration ratio, *I* represents the incident solar flux density measured in [W/m^2^], σ defines the Stefan–Boltzmann constant, and *T* and *T*_0_ are the receiver and ambient temperature [K], respectively. In this work, we used the following parameters for the calculation of η(*T*): *C* = 100 and 1000;^39^*I* = 892 W/m^2^; *T* = 600 °C (873 K), 700 °C (973 K), and 800 (1073 K); *T*_0_ = 25 °C (298 K).

The performance criterion (PC), that can be obtained evaluated by adding the changes in absorptance (Δα) and emittance (Δε) using the equation:^40^
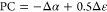
4where Δα = α (aged) – α (unaged) and Δε = ε (aged) – ε (unaged). A maximum performance decrease of PC = 0.05 (5%) is considered acceptable.^40^

## 3 Results and Discussion

Figure 1 depicts a scheme of the targeted SSC architecture. The coating structure and nominal chemical composition of this solar selective absorber previously presented in ref (32) are as follows (from bottom to top): a Cr_0.96_Al_0.04_N_0.89_ layer that serves both as an IR reflector and solar absorber, a Cr_0.62_Al_0.38_N_1.00_ film as a semiabsorber layer, followed by a Cr_0.47_Al_0.53_N_1.12_ film to reduce gradually the refractive index up to the antireflective Al_2_O_3_ top-layer.

The chemical composition was first assessed by RBS on individual single layers grown on silicon in identical conditions as used in this stack grown with bias assistance. The stoichiometries obtained experimentally are summarized in Table 3 in comparison with the nominal values. Further information on the RBS spectra and simulation can be found in the Supporting Information (Figure S1). In general, the obtained nitride stoichiometries follow the expected metal and nitrogen concentration with a substoichiometric C1 layer (with N vacancies and small Al content) used as an IR reflector and absorber layer^31^ followed by two layers with growing Al contents. The aluminum oxide is slightly overstoichiometric with a density of 2.7 ± 0.1 g/cm^3^, *n* ≈ 1.6, and *k* = 0 in the UV–vis–NIR range.

**Table 3:** Individual Film Stoichiometries Determined by RBS and TEM

code	nominal	measured thickness, nm	experimental RBS/TEM-SS316/TEM-INC625
C1	Cr_0.96_Al_0.04_N_0.89_	88	Cr_0.95_Al_0.05_N_0.95_
			Cr_0.92_Al_0.08_N_0.79_
			Cr_0.92_Al_0.08_N_0.83_
C2	Cr_0.62_Al_0.38_N_1.00_	18	Cr_0.71_Al_0.29_N_0.98_
			Cr_0.63_Al_0.37_N_1.12_
			Cr_0.64_Al_0.36_N_1.07_
C3	Cr_0.47_Al_0.53_N_1.12_	35	Cr_0.51_Al_0.49_N_1.03_
			Cr_0.44_Al_0.56_N_1.16_
			Cr_0.46_Al_0.54_N_1.14_
Al–O	Al_2_O_3_	91	Al_2_O_3.4_
			Al_2_O_3.16_
			Al_2_O_3.80_

The chemical composition of the SSCs was also measured by EDX analysis on TEM cross sections for the SS316 and INC625 substrates (cf. Table 3). The nature of the metallic substrate did not affect the atomic chemical compositions of the individual nitride layers measured by TEM as the obtained stoichiometries are almost identical. A good agreement can be found between RBS and EDX/TEM, although the layer stoichiometries do not coincide exactly. It should be noted that RBS measurements were done on silicon vs metallic substrates in the latter case.

**Figure 2:**
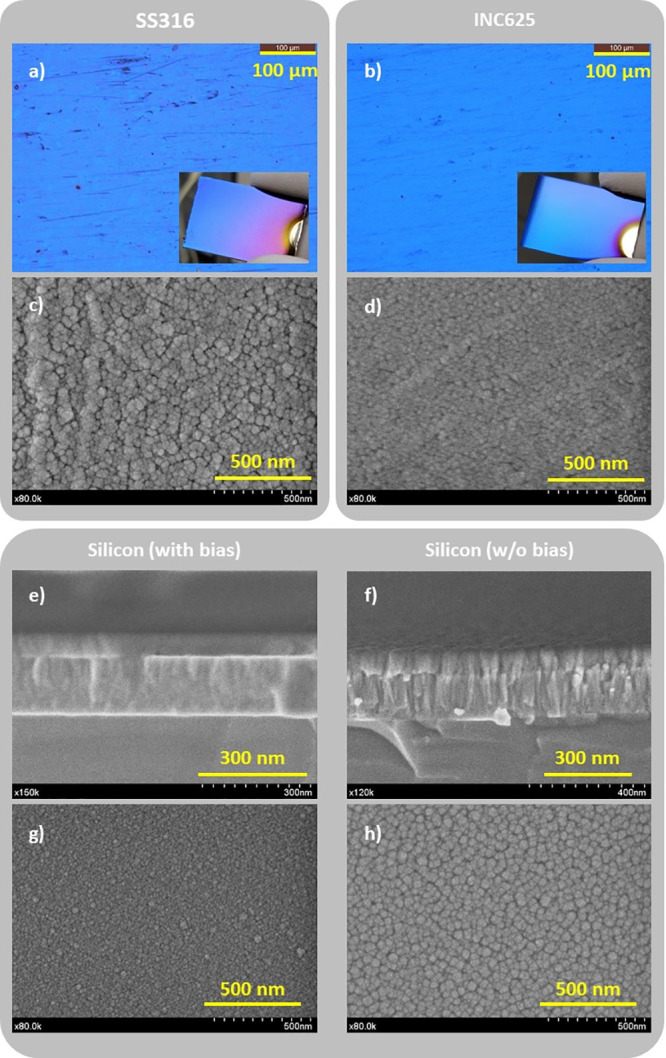
Optical and top-view scanning electron micrographs taken from the surface of the stacks deposited on SS316 (a, c) and INC625 (b,d). In the bottom part, the pictures correspond to the cross-section and top views of the stack grown on silicon using bias (e,g) and without bias (f,h), respectively.

Figure 2 includes selected micrographs taken on the stacks deposited on SS316, INC625, and silicon substrates. The optical micrographs and visual aspects of the stacks deposited on the metallic specimens are shown in Figure 2a and b. The presence of surface heterogeneities is clearly observed, as the substrates were not mirror-polished. The coated specimens display a violet-blue color within the range of SSCs. The top view analysis (Figure 2c and d) exhibits the typical structure formed by the dome of the columns, with a mean diameter that decreases from 55 nm for the stainless steel to 45 nm for the Inconel. This trend results in agreement with the initial surface finishing of the substrates (RMS = 62 and 42 nm, respectively). In the bottom part, the film microstructure and top surface of the stack deposited on silicon with and without bias assistance are comparatively assessed. The cross-sectional views obtained by scanning electron microscopy (cf. Figure 2e and f) reveal clearly the bilayer (nitride/oxide) structure in both cases. However, the comparison of the microstructure of the layers that form part of this stack demonstrates that both the nitride and oxide sections of the biased sample are less columnar thanks to the additional bombardment provided during growth. The analysis of the top views confirmed the greater compactness and smaller column diameters of the biased sample. A more detailed microstructural and chemical analysis is then carried out by examining a thin lamella of the SSC grown directly on the INC625 substrate.

**Figure 3:**
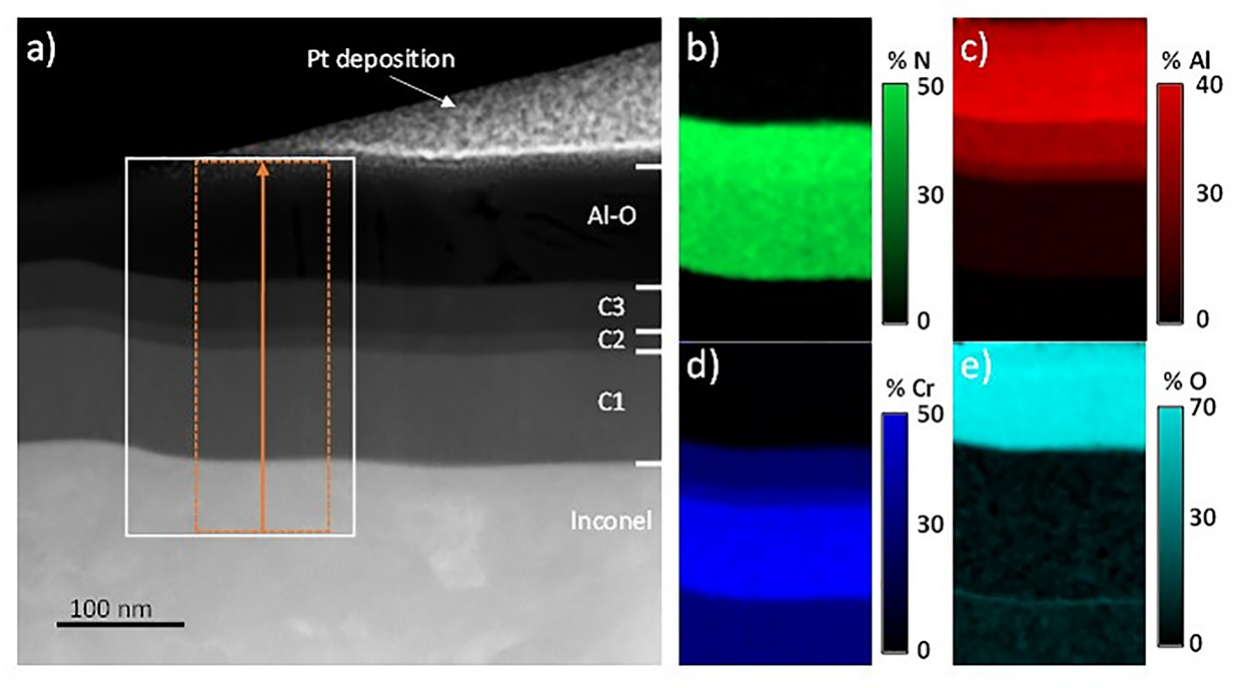
(a) HAADF image of INC625 sample and (b–e) EDX elemental map of N, Al, Cr, and O performed on a white ROI of image a.

Figure 3 depicts an HAADF-STEM or Z-contrast image of the stack deposited on INC625 together with the associated EDX atomic element distribution maps for a selected cross-section region marked in white. The four different layers composing the stack can be clearly observed with increasing contrast. In the HAADF-STEM technique, in opposition to bright field TEM, brighter zones correspond to a higher density and atomic numbers (*Z*). Therefore, the contrast gradient is related to an increasing content of aluminum in the nitride layers (Al has a lower *Z* than Cr) and the top aluminum oxide layer. The elemental maps corroborate this atomic distribution, as can be concluded from Figure 3b to e. The thickness of the different layers forming the stack can be measured directly in the HAADF image in good agreement with the design proposed in Figure 1.

**Figure 4:**
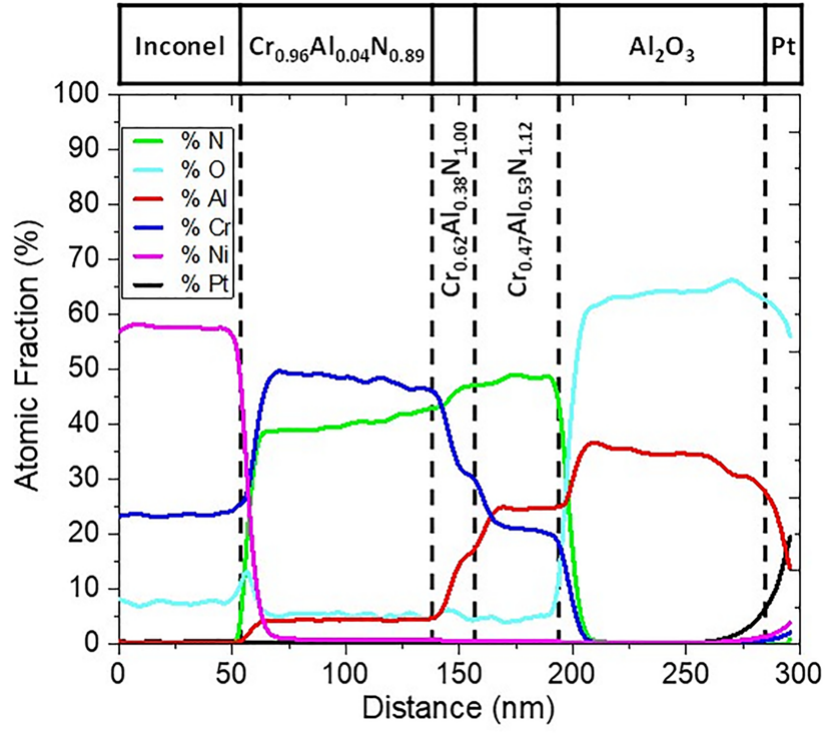
Elemental composition line profile obtained along a cross-section of the INC625 sample from the orange square marked in Figure 3a.

Figure 4 shows the EDX elemental composition line profiles across the multilayered stack grown on INC625 in the direction marked by the arrow. Table 3 summarizes the measured layer thicknesses from these line profiles. The layer interfaces appear to be clearly defined except for the C2 layer. Due to its limited thickness, the chemical composition did not reach a steady state, displaying a continuous change. The nitrogen profile is continuously increasing up to C3 where it stabilizes around 50 atom %. As expected, the nitrogen content is found to be around 40 atom % in C1 in order to generate N vacancies. The oxygen level is found to be lower than 10 atom %, both in the nitride layers and in the Inconel substrate, which indicates that this oxygen might be incorporated during the preparation of the thin film lamella due to its limited thickness (80–100 nm) after exposure to air. The Al–O top layer is well distinguished with a slightly overestimated Al/O ratio of 35:65, which can also be influenced by oxygen incorporation during TEM preparation.

**Figure 5:**
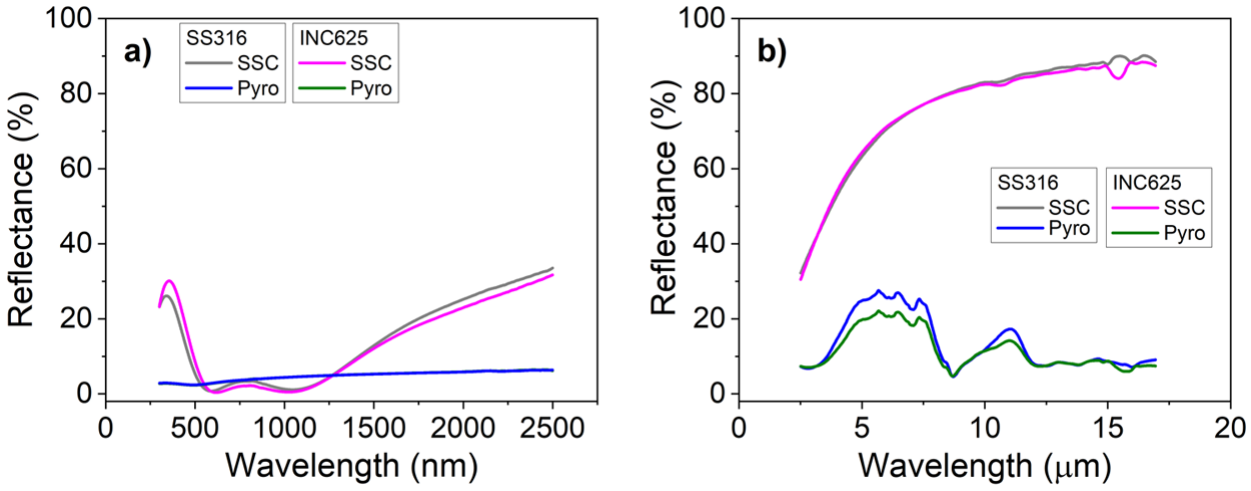
Reflectance spectra of the stacks deposited on SS316 and INC625 compared with Pyromark in the UV–vis–NIR (a) and IR (b).

Figure 5a and b depict the reflectance spectra for the stacks grown on SS316 and INC625 in UV–vis–NIR and Mid-IR, respectively. The curves obtained for Pyromark coated specimens are also included for comparison purposes. The calculated absorptance and emissivity values for these curves were calculated according to eqs 1 and 2 giving an average of α = 94.7 and ε_25°C_ = 18% for the SS316 and α = 94.3 and ε_25°C_ = 18% for the INC625. The α and ε values are slightly lower and higher respectively than those measured at 25 °C for the stack grown without bias (α = 96 and ε_25°C_ = 15%).^32^ This can be attributed to the compositional changes in the C1 layer and a variation of the optical properties due to the increased film density achieved with the assistance of bias. A slightly higher aluminum content and a reduction of the nitrogen vacancies in the first layer of the stack could decrease the metallic-like character, reducing the solar absorptance and the IR reflectivity. The increment in the layer density leads to higher refraction indexes that can modify the optical behavior of the stack.

**Figure 6:**
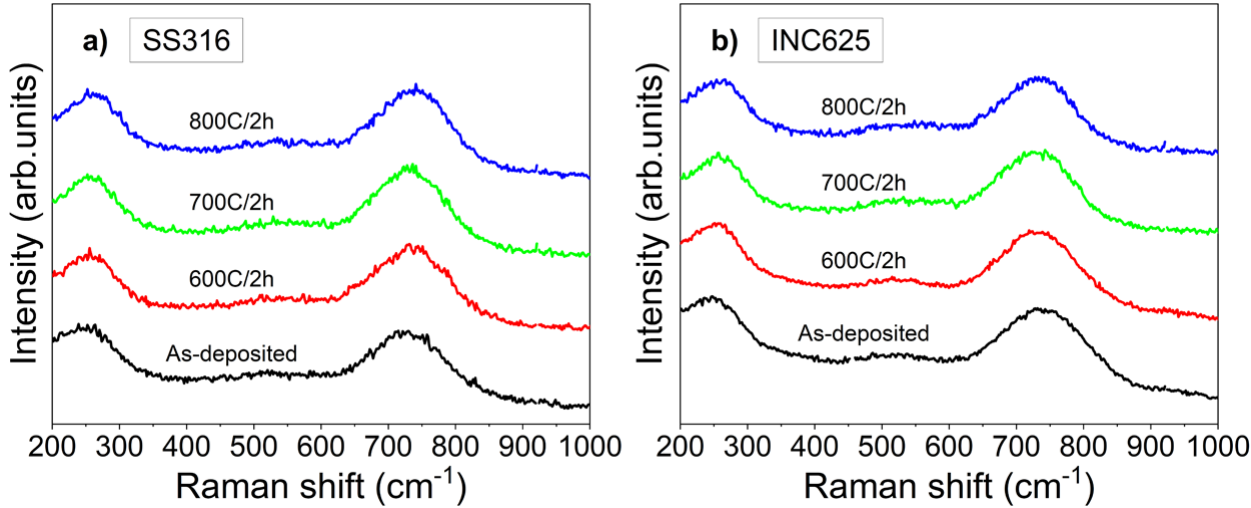
Raman spectra of the as-deposited and annealed stacks on SS316 (a) and INC625 (b).

The multilayered stacks deposited on both metallic substrates were heated in air at 600, 700, and 800 °C for 2 h to study the thermal stability and modifications of the optical performance. The initial Raman spectra for the SSC stacks deposited on both types of substrates and after annealing at each temperature are presented in Figure 6. The most significant finding is that the spectra remain unchanged, with the presence of broad bands at 250 and 700 cm^–1^, resulting from acoustic and optic phonon vibrations in defective nonstoichiometric Cr(Al)N. Similar analysis carried out in the stack grown without bias in paper^32^ put in evidence a gradual shift of these phonon bands toward higher values at 600 and 700 °C and the onset of oxidation at 800 °C. The shift toward higher frequencies was found to be correlated with an increase in the aluminum content in the outer layers and/or the presence of nitrogen vacancies induced during heating.^41,42^ At 800 °C, the apparition of a tail in the region between 500 and 700 cm^–1^ was associated with a sum of various chromium oxides, Cr_2_O_3_, Al_*x*_Cr_2–*x*_O_3_, and CrO_2_.^43,44^ However, the absence of these features in this case demonstrates a higher oxidation resistance achieved by the additional ion bombardment provided by bias assistance.

**Figure 7:**
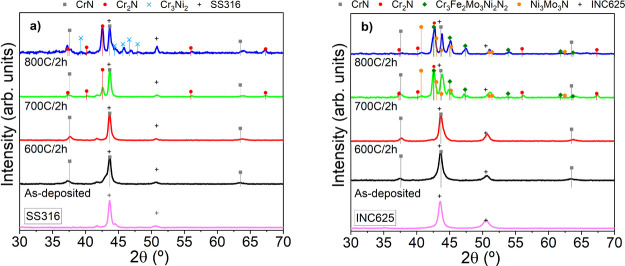
XRD diffractograms of the as-deposited and annealed stacks on SS316 (a) and INC625 (b). JCPDS cards numbers: CrN (PDF #76-2494); Cr_2_N (PDF #35-803); Cr_3_Ni_2_ (PDF #26-430); SS316 (PDF #35-1375); Cr_3_Fe_2_Mo_3_Ni_2_N_2_ (PDF #26-428); Ni_3_Mo_3_N (PDF #49-1336); INC625 (PDF #35-1489).

Figure 7 illustrates the changes in the grazing XRD diffractograms of the initial specimens and after the annealing measured in the region of 30° to 70°. The initial scans only display the reflection peaks corresponding to the CrN phase together with the peaks originated by the substrates. No significant changes are observed up to 700 °C with the appearance of new peaks. The formation of Cr_2_N is manifested by the increase in a peak at 42.6° corresponding to the main reflection (111) of this hexagonal phase. The formation of this new phase proceeds by the release of nitrogen from the nitride layers activated by the increase of temperature, outward metal ions diffusion from the substrate, and inward oxygen penetration. However, this thermal decomposition proceeds 100 °C later than the same stack grown without bias,^32^ where this phase is already present at 600 °C. Comparing both types of substrates, it is clearly observed that ion interdiffusion and thermal decomposition proceeds to a great extent in the INC625. The most intense new peaks correspond to intermetallic nitrides, including different ions from the substrate (Cr_3_Fe_2_Mo_3_Ni_2_N and Ni_3_Mo_3_N). These phases are formed between the nitrogen released by thermal decomposition and subsequent reaction with the metallic elements diffusing from the substrate. The formation of Cr_2_N phase is also inferred, although the main reflection overlaps with the maximum of the Cr_3_Fe_2_Mo_3_Ni_2_N intermetallic phase from the presence of peaks at 56 and 67°, reflections (112) and (300), respectively. The increase up to 800 °C brought the noticeable increment of the Cr_2_N phase in the SS316 and Cr_3_Fe_2_Mo_3_Ni_2_N in the INC625. These results are pointing out the relevance of the type of substrate in the chemical transformation that the multilayered stack suffered during heating in air. A similar conclusion was highlighted in our previous publication regarding the oxidation and diffusional processes in steel coupons protected with CrAl(Y)N coatings annealed at a high temperature.^45^ There is no evidence of incipient Cr_2_O_3_ and Al_2_O_3_ peaks as they were observed in the stack grown without bias on SS316 at 800 °C. These results are indicative of a higher protective character of the alumina top layer and/or higher oxidation resistance of the CrAlN in correlation with the less columnar microstructure demonstrated by microscopy analysis.

**Figure 8:**
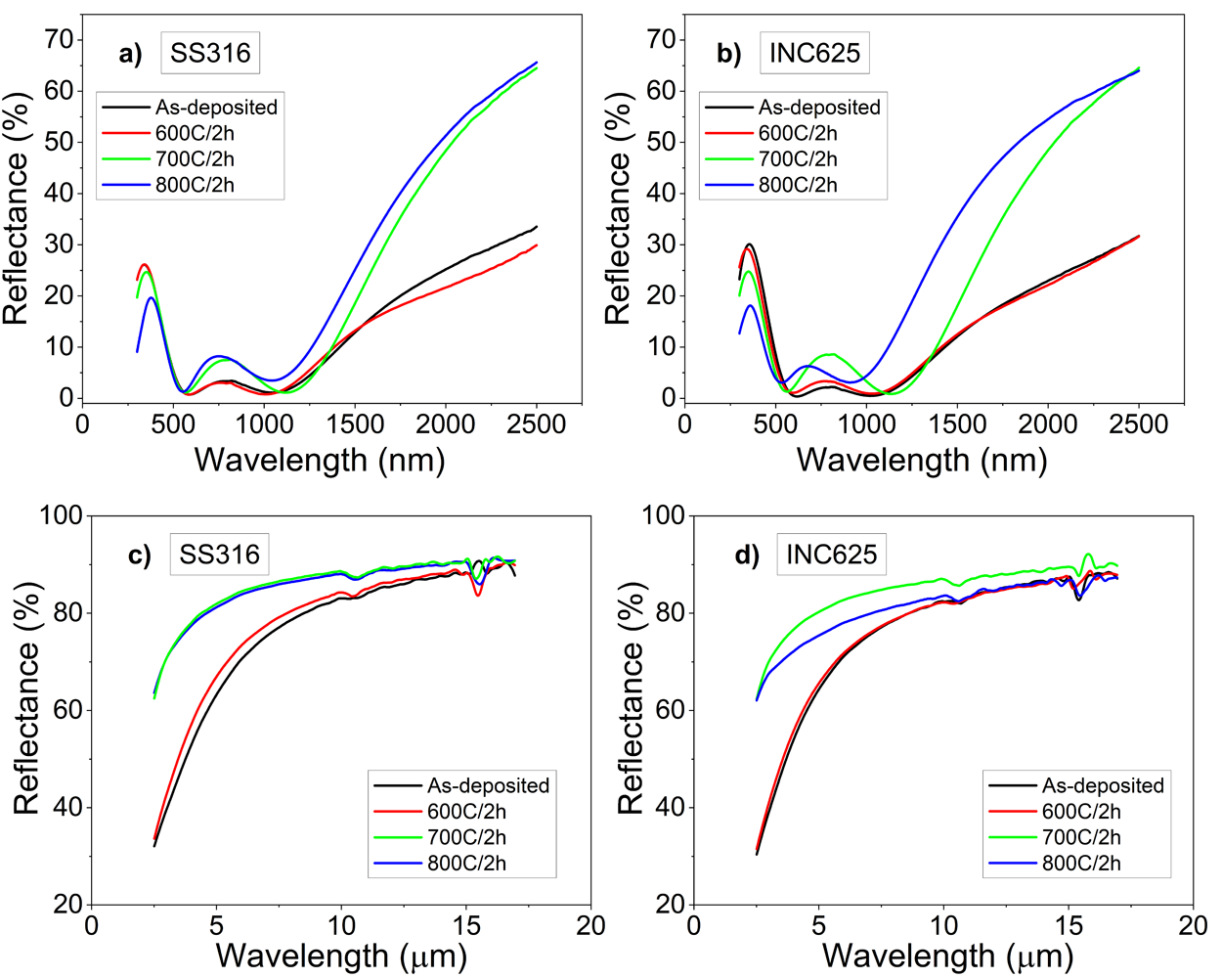
Reflectance spectra of the as-deposited and annealed stacks on SS316 and INC625 at 600, 700, and 800 °C over 2 h in air in the UV–vis–NIR (a,b) and IR (c,d).

**Figure 9:**
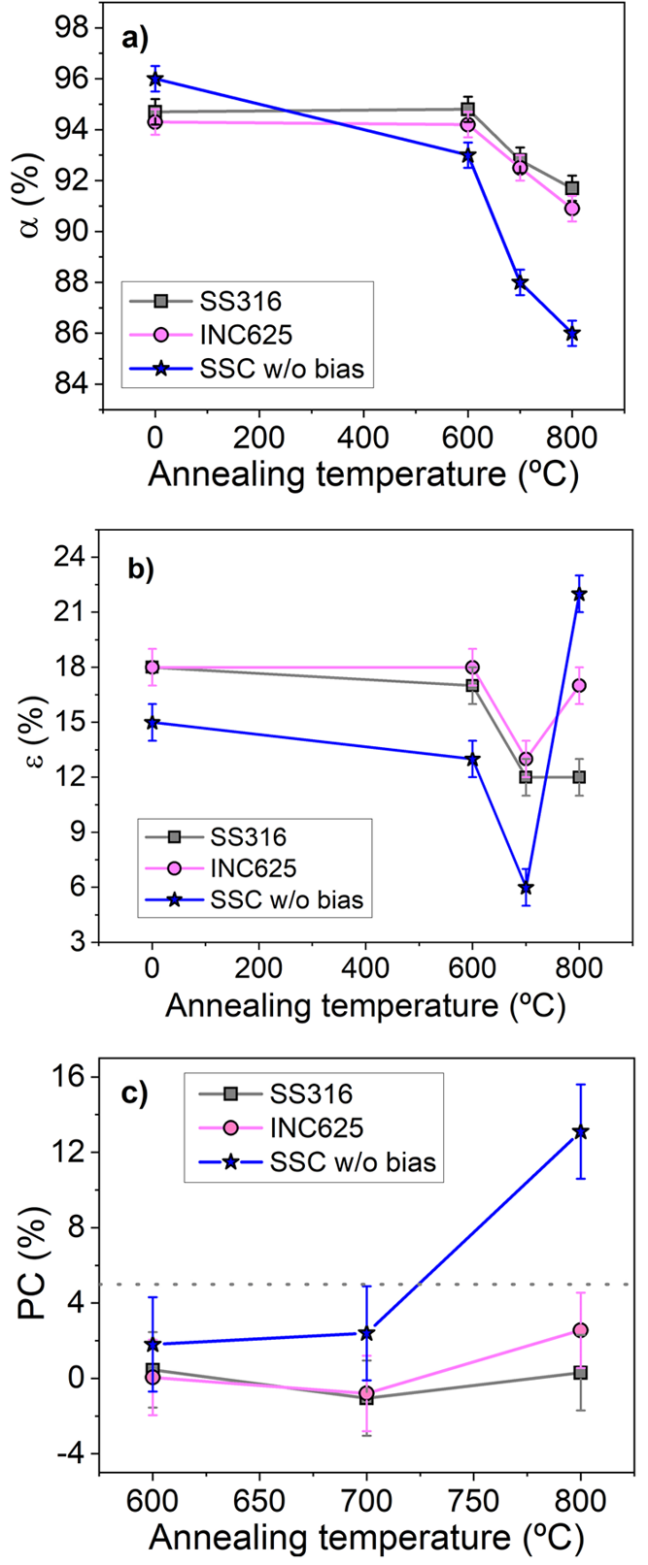
Evolution of the (a) solar absorptance α, (b) thermal emittance ε, and (c) performance criterion PC, measured after the single-stage thermal tests at 600, 700, and 800 °C of the SSCs prepared in this work using bias on SS316 and INC625 substrates, in comparison with the behavior exhibited by SSC without bias on SS316.

Figure 8 displays the reflectance spectra obtained at each temperature, presented comparatively to the initial spectrum. At 600 °C, the spectra for both types of substrates are very similar to those obtained at room temperature, in good agreement with the XRD analysis where the changes started at 700 °C. At this temperature, the intensity of the band centered at 800 nm slightly grows and the reflectance at 2500 nm rises up to 60%. At 800 °C, although the main bands at ∼365 nm decrease, the shift of the edge of the NIR high reflectance region toward lower wavelengths (particularly in the case of INC625) indicates a deterioration of the desired optical performance. In the IR region (cf. Figure 8b), an increment of the reflectance is observed above 700 °C for both substrates, which has a positive influence on the decrease of the IR emissivity losses. The corresponding figures for the Pyromark references are reported in the Supporting Information (Figure S2). Figure 9 depicts the evolution of the optical parameters (α and ε) and the performance criterium (PC) vs the annealing temperature in comparison with the analogous stack grown without bias. The evolution of α (cf. Figure 9a) clearly demonstrates that the use of bias has introduced a noticeable improvement in the thermal stability of the stack, showing higher values upon annealing. The absorptance values are maintained up to 600 °C at approximately 95% and decrease to 92–93% at higher temperatures, whereas the stack grown without bias suffered a strong decrease at 700 °C to 88% and continued toward 86% at 800 °C. Comparable α values, within the deviation bars, are obtained for both substrates after annealing at these temperatures, whereas different behavior is observed in the case of emittance. For SS316, the emittance values manifested a significant improvement at 700 and 800 °C, as they are still lower than the initial values. The decrease in emittance is correlated with the increase in the metallic character originating from the formation of the Cr_2_N phase, which is promoted by thermal decomposition of the most unstable Cr_0.96_ Al_0.04_N _0.89_ bottom layer. A similar behavior is observed in the stack grown without bias but degrades significantly at 800 °C. In the case of INC625, the emittance at 800 °C is higher than the stack deposited on the steel substrate. This difference can be correlated with the remarkable ion interdifussion observed at 800 °C by XRD analysis. In Figure 9c, the performance criterium figure of merit reveals that the SSC coatings grown with the assistance of bias are significantly better than the reference one, with values below 5% at all temperatures. Table 4 summarizes the solar absorptance and thermal emittance values at 25 and 600 °C for the as-deposited and annealed stacks in comparison with those obtained for Pyromark reference measured after similar thermal treatment.

**Table 4:** Solar Absorptance and Thermal Emittance Values at 25 and 600 °C for the As-Deposited and Annealed Stacks (600, 700, or 800 °C for 2 h) in Comparison with Those Obtained for Pyromark Reference Measured after Similar Thermal Treatment

		as deposited			600 °C			700 °C			800 °C		
substrate		α	ε_25°C_	ε_600°C_	α	ε_25°C_	ε_600°C_	α	ε_25°C_	ε_600°C_	α	ε_25°C_	ε_600°C_
SS316	SSC	94.7	18	42	94.8	17	39	92.8	12	23	91.7	12	23
	Pyromark	96.4	87	85	96.6	88	86	96.6	91	89	96.0	90	88
INC625	SSC	94.3	18	42	94.2	18	41	92.5	13	24	90.9	17	27
	Pyromark	96.4	89	87	96.7	92	91	96.6	91	90	96.1	89	87

**Figure 10:**
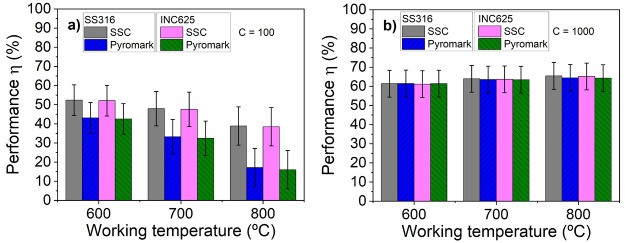
Calculated solar performances η of the as-deposited solar selective stack on both substrates (SS316 and INC625) in comparison with Pyromark at target working temperatures of 600, 700, and 800 °C for concentration factors *C* = 100 (a) and *C* = 1000 (b).

The solar performance (η) of this stack was evaluated using eq 3 for two concentration factors (*C*) of 100 and 1000. The obtained results were then compared with the performance of Pyromark at target working temperatures of 600, 700, and 800 °C. The obtained results are plotted in Figure 10a and b. At a concentration factor of *C* = 100, the performance of the stack shows a remarkable improvement compared to Pyromark, even above 700 °C, where structural and chemical transformations begin. Specifically, at a target working temperature of 700 °C, the solar performance of the deposited stack is approximately 15 percentage points higher than the Pyromark efficiency. Furthermore, at 800 °C, the solar performance of the stack is more than double that of the Pyromark absorber commercial paint. In other words, the selective stacks are able to achieve the same efficiencies as Pyromark but at lower concentration factors. This behavior results independently on the type of substrate, although SS316 is slightly better than INC625. At higher concentration factors (see Figure 10b for *C* = 1000), the performance of the SSC stacks becomes comparable to that of Pyromark.

**Figure 11:**
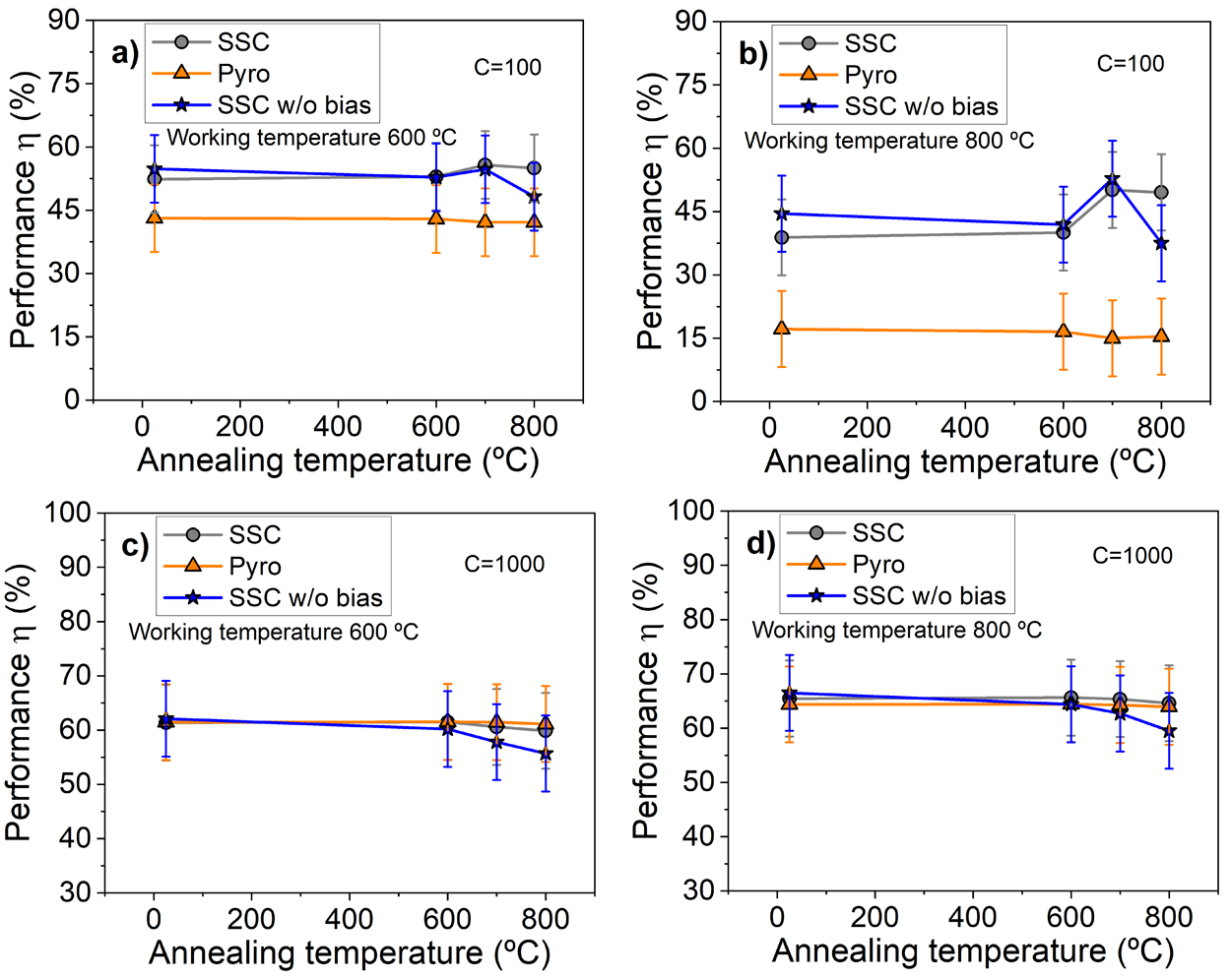
Evolution of the solar performances η of the annealed stacks in comparison with Pyromark and SSC deposited without bias on SS316 calculated at target working temperatures of 600 and 800 °C at two concentration factors: *C* = 100 (a,b) and *C* = 1000 (c,d).

**Figure 12:**
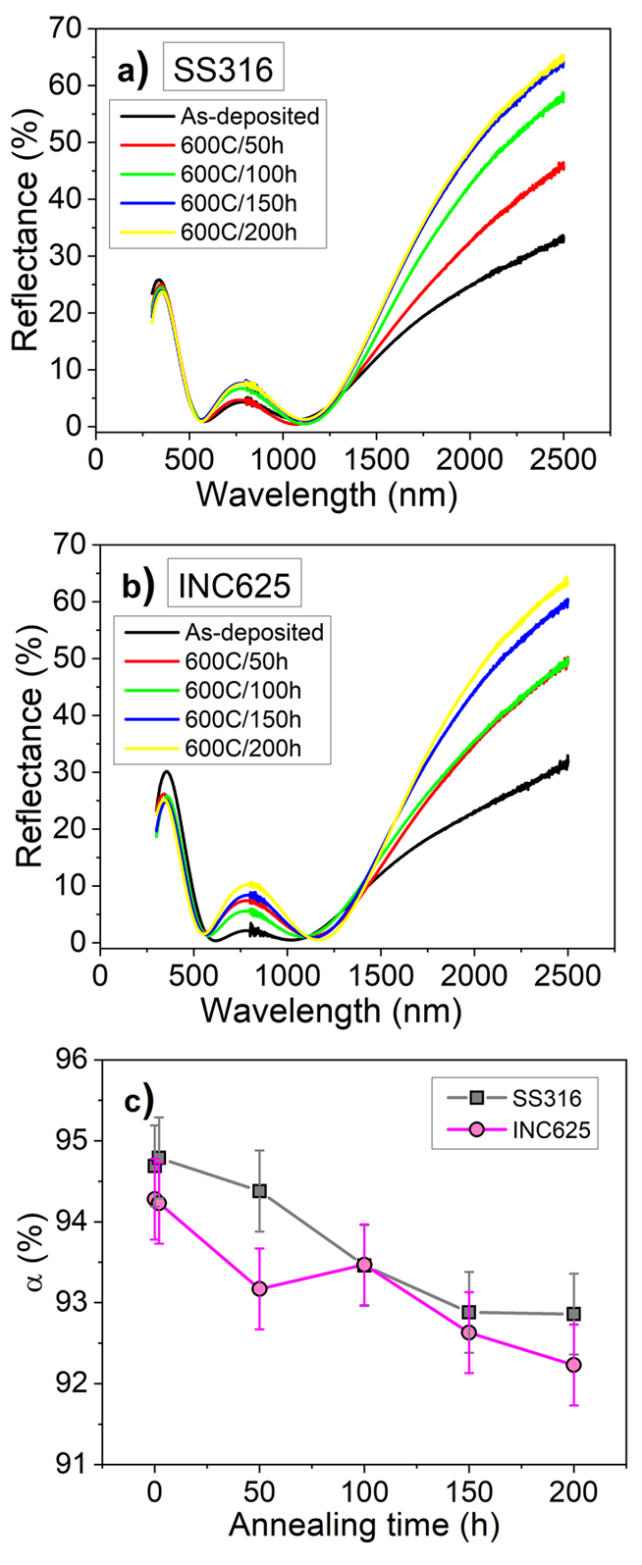
Reflectance spectra of the as-deposited and annealed stacks at 600 °C over 50, 100, 150, and 200 h in air in the UV–vis–NIR on SS316 (a) and INC625 (b) and time evolution of the solar absorptance α (c).

**Figure 13:**
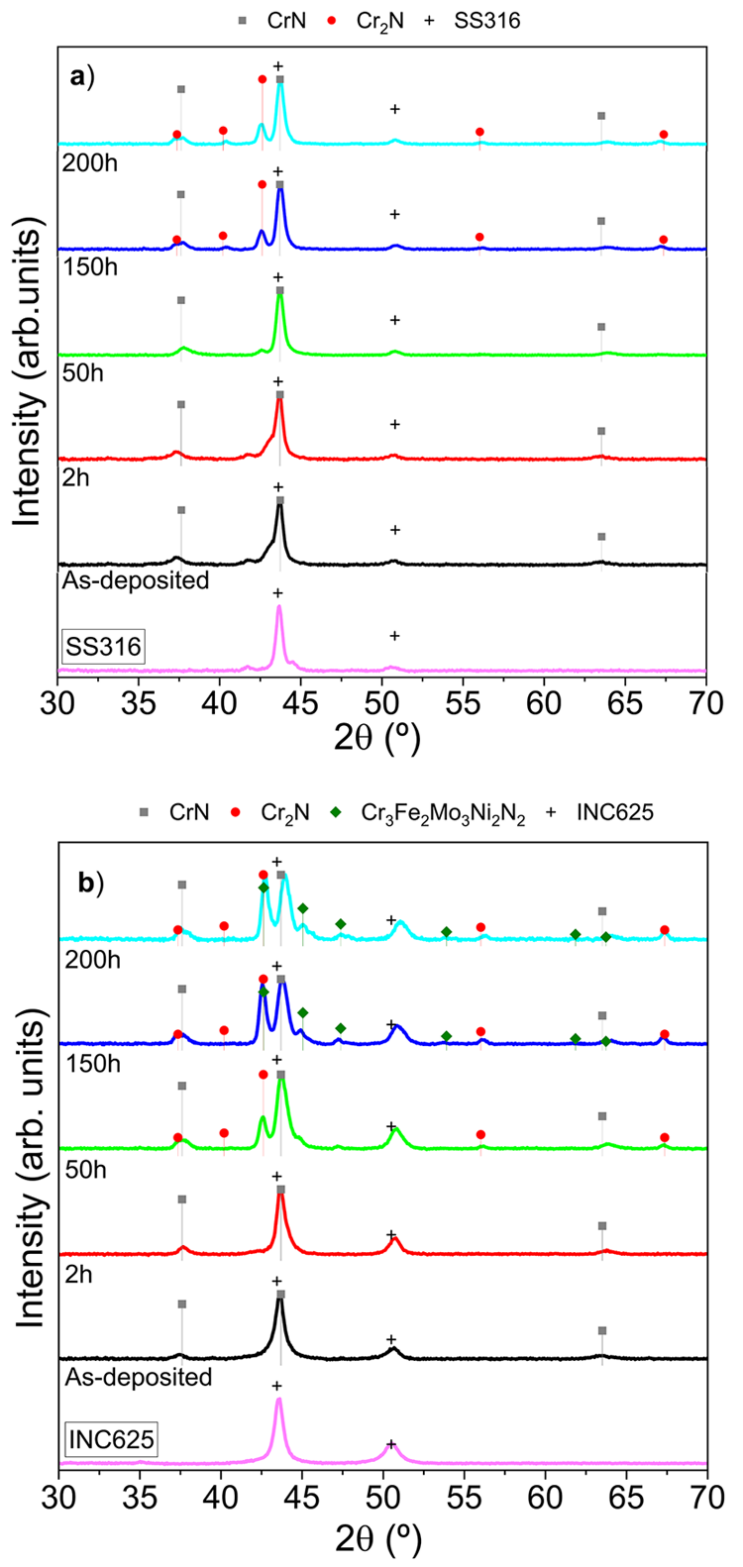
XRD diffractograms of the as-deposited and annealed stacks on SS316 (a) and INC625 (b) at increasing intervals from 2 to 200 h at 600 °C. JCPDS cards numbers: CrN (PDF #76-2494); Cr_2_N (PDF #35-803); SS316 (PDF #35-1375); Cr_3_Fe_2_Mo_3_Ni_2_N_2_ (PDF #26-428); INC625 (PDF #35-1489).

A comparison between the annealed stacks at the different working temperatures versus Pyromark specimens and the homologous stack grown without bias would yield insightful results. Figure 11 shows the evolution of η of the annealed stacks deposited on SS316 at target working temperatures of 600 and 800 °C for concentration factors *C* = 100 (Figure 11a and b) and *C* = 1000 (Figure 11c and d). We have selected the coated steel substrates to allow direct comparison with the stacks deposited without bias reported in ref (32). The corresponding plot at 700 °C is very similar to that of 600 °C and was moved to the Supporting Information together with the set of SSCs deposited on INC625 (Figures S3 and S4). At *C* = 100, independently of the used substrate, outstanding performance values of between 40 and 55% are obtained, which are 15–35% points higher than Pyromark efficiency at these working temperatures, respectively. The reduction of emittance from 700 °C compensates for the reduction in the α values. The improved thermal behavior of the current SSC deposited with bias is manifested particularly at 800 °C where the performance is maintained. At high concentration factors (cf. Figure 11c and d) the performance of the SSC becomes comparable to that of Pyromark but still better than that of the SSC grown without bias. Longer annealing times (up to 200 h in intervals of 50 h) were carried out to check the stability at a much longer extent. The evolution of the reflectance spectra and the absorptance values at 600 °C for the SSC on the two types of substrates is plotted in Figure 12. The obtained final (α and ε) values at 200 h are about 93 and 92% for SS316 and INC625, and the corresponding measured emittance values are 12 and 13%, respectively. With these results the PC values are still below zero in both cases. The corresponding XRD analysis (shown in Figure 13) demonstrated that the presence of Cr_2_N is significant in INC625 from 50 h onward, while a similar pattern started in SS316 at 150 h. Similar to the annealing process at 800 °C for 2 h, the INC625 manifested the formation of intermetallic nitrides including different ions from the substrate from 150 h. The XRD diagrams at 200 h resemble those of the previous heating stage with no evidence of metal oxides from substrate or SSC components, indicative of a good thermal stability and oxidation resistance at this temperature. The calculated solar-to-mechanical performance after 200 h for *C* = 100 revealed that η ≥ 50% for all temperatures and better than the initial ones (cf. Figure S5 Supporting Information), proving the excellent optical performance. Nevertheless, longer validation tests and sun exposure in solar furnaces at 650 °C are currently in progress to check the present results.

## 4 Conclusions

A multilayered CrAlN_1–*x*_/CrAl(Lo)N/CrAl(Hi)N/Al_2_O_3_ was grown via a combination of magnetron sputtering technologies (HiPIMS, RF, and DC-p) with simultaneous biasing of the substrates with the aim of increasing the high temperature stability and spectral performance for solar receivers. The performance of the SSC stacks grown onto two types of metallic substrates (316L stainless steel and Inconel 625) was studied and compared with a similar stack grown without bias and Pyromark painting as a reference. The initial stacks displayed α ≈ 95% and ε ≈ 18% (calculated for 25 °C) independently of the substrate. After annealing at 600, 700, and 800 °C in air over 2 h, the absorptance values showed a progressive decrease, which is compensated for by a reduction in IR losses. The origin behind these optical changes is related to the partial transformation of the CrAlN to Cr_2_N and intermetallic nitrides formed with the ions delivered by the Inconel substrate, as confirmed by XRD analysis. The as-prepared and annealed stacks at 600, 700, and 800 °C stacks demonstrated superior solar-to-mechanical energy conversion efficiency (η) compared to Pyromark (at *C* = 100) and a comparable (at *C* = 1000) but much better one than the SSC grown without bias. The solar performance is found to be slightly lower when using Inconel 625, particularly when annealing at 800 °C. Nevertheless, both SSCs fulfilled the performance criterium even at 800 °C while the homologous stack grown without bias overpassed the 5% limit. Longer annealing times up to 200 h in air were tested at 600 °C, showing that chemical transformation and optical properties stabilize (particularly in the case of SS316), with solar performances higher than 50–60%, for concentration factors *C* = 100 to 1000. The additional ion bombardment provided by the bias assistance has led to an increment of the lifetime, thermal stability and working limit temperature as compared to similar unbiased coatings, thanks to a more compact microstructure. Further investigations are ongoing to check the practical performance under high fluxes in solar furnaces and the design of the barrier diffusion layer over the substrates.

## Supporting Information Available

Details on the RBS characterization of the samples; reflectance spectra of the as-deposited Pyromark before and after annealing in air; evolution of the solar performances of the annealed stacks on both types of substrates calculated at target working temperatures of 600, 700, and 800 °C at two concentration factors (*C* = 100 and 1000); evolution of the solar performances of the annealed stacks in comparison with Pyromark and similar stacks deposited without bias on SS316 calculated at 700 °C at two concentration factors (*C* = 100 and 1000) (PDF)
