# Supplementary material for: Synthesis and Characterization of Multilayered CrAlN/Al2O3 Tandem Coating Using HiPIMS for Solar Selective Applications at High Temperature
Source: ACS Appl Energy Mater. 2023 Dec 29;7(2):438–49. doi: 10.1021/acsaem.3c02310 (PMC10806925; doi:10.1021/acsaem.3c02310)

SUPPLEMENTARY MATERIAL

Synthesis and characterization of multi-layered CrAlN/Al_2O_3 tandem coating using HiPIMS for solar selective applications at high temperature

Miriam Sanchez-Perez[1], *Teresa Cristina Rojas*[1], *Daniel F. Reyes*[1,2], *F. Javier Ferrer*[3,4], *Meryem Farchado*[5], *Angel Morales*[5], *Ramon Escobar-Galindo*[6], *Juan Carlos Sanchez-Lopez*[1,*]

[1] Instituto de Ciencia de Materiales de Sevilla (CSIC-Univ. Sevilla), Avda. Américo Vespucio 49, E-41092 Sevilla, Spain

[2] University Research Institute on Electron Microscopy & Materials, (IMEYMAT), Universidad de Cádiz, E-11510 Puerto Real (Cádiz), Spain

[3] Centro Nacional de Aceleradores (Univ. Sevilla, CSIC and Junta de Andalucía), Avda. Tomás A. Edison 7, E-41092 Sevilla, Spain

[4] Departamento de Física Atómica, Molecular y Nuclear, Universidad de Sevilla, Aptdo 1065, E-41012 Sevilla, Spain

[5] CIEMAT-PSA, Materials for Concentrating Solar Thermal Technologies Unit, Avenida Complutense 40, E-28040 Madrid, Spain

[6] Departamento de Física Aplicada I, Escuela Politécnica Superior, Universidad de Sevilla, Virgen de África 7, E-41011, Sevilla, Spain

* E-mail: jcslopez@icmse.csic.es

Fig. S1: RBS spectra at low (a) 1.0 MeV (C1 and C3) and 2.0 MeV (C2) and high energy (b) 3.7 MeV for all the samples.

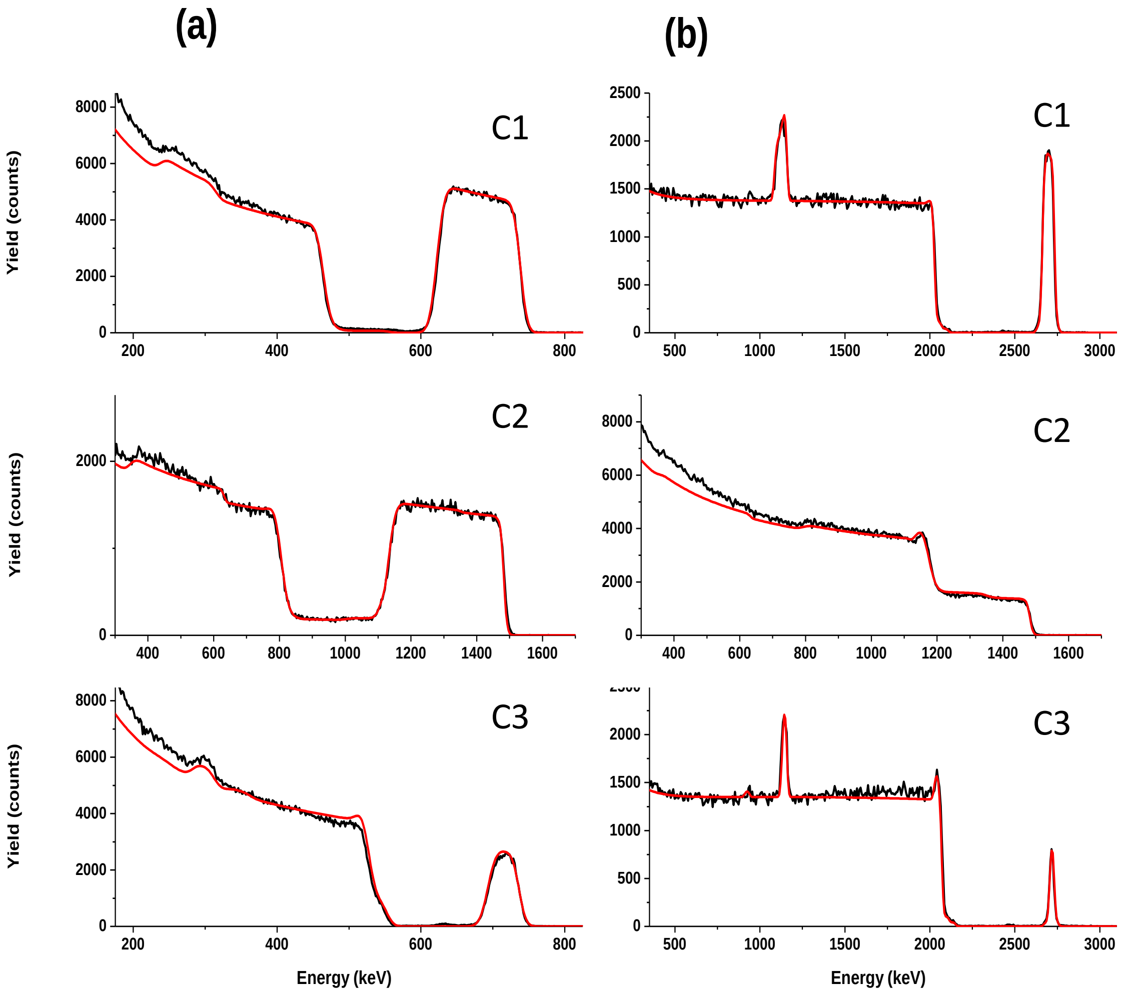

	Thickness (10^{15} at/cm^2)	Cr	Al	N
C1	1019 ± 58	0.95 ± 0.01	0.05 ± 0.01	0.95 ± 0.01
C2	3760 ± 256	0.71 ± 0.01	0.29 ± 0.01	0.94 ± 0.01
C3	423 ± 34	0.51 ± 0.01	0.49 ± 0.01	1.03 ± 0.01

Fig. S2: Reflectance spectra of the as-deposited Pyromark and annealed during 2 hours in air in the UV-vis-NIR (a, b) and IR (c,d).

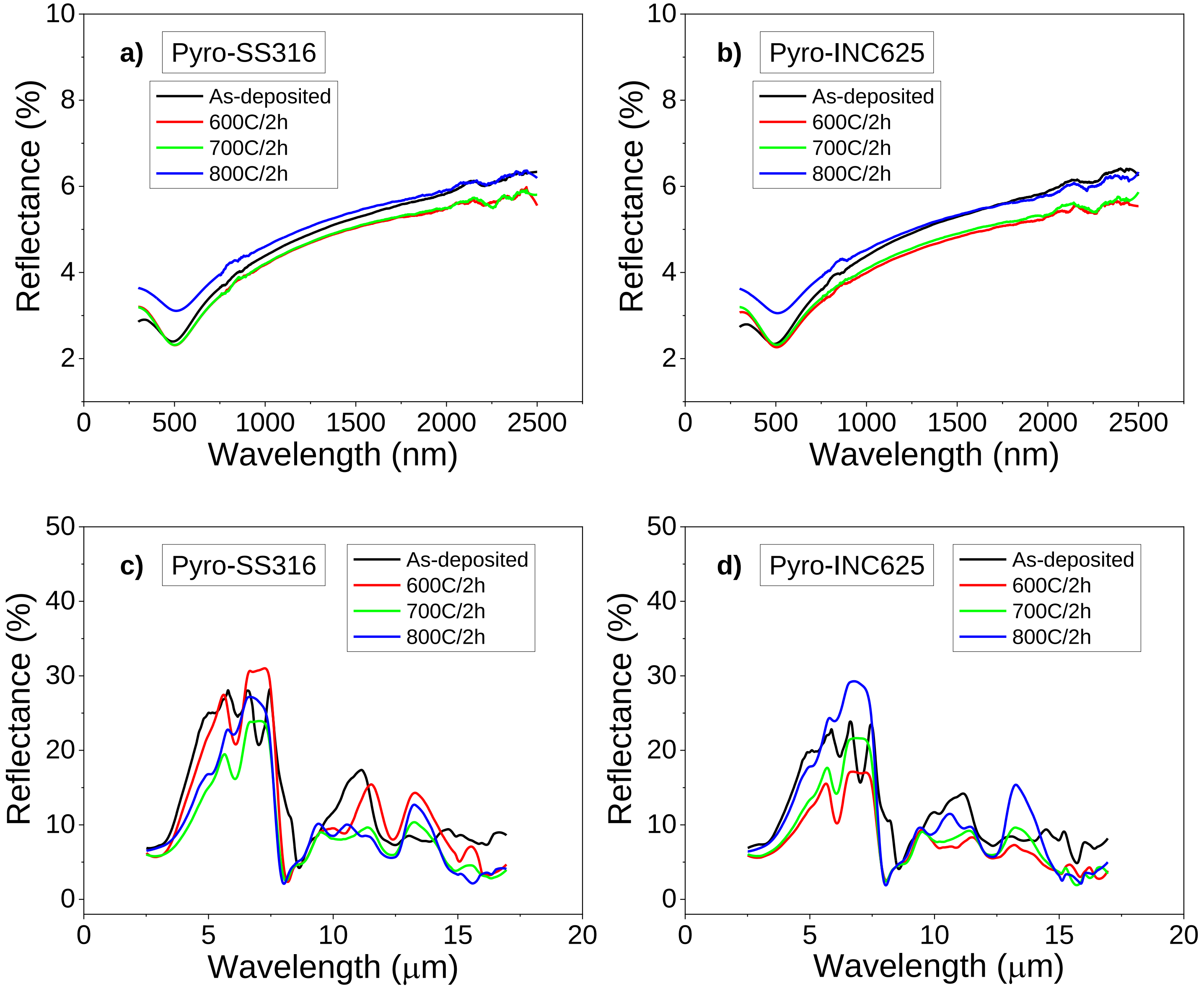

Fig. S3: Evolution of the solar performances η of the annealed stacks in comparison with Pyromark and SSC deposited without bias on SS316 calculated at target working temperatures of T = 700 °C at two concentration factors: C = 100 (a) and C = 1000 (b).

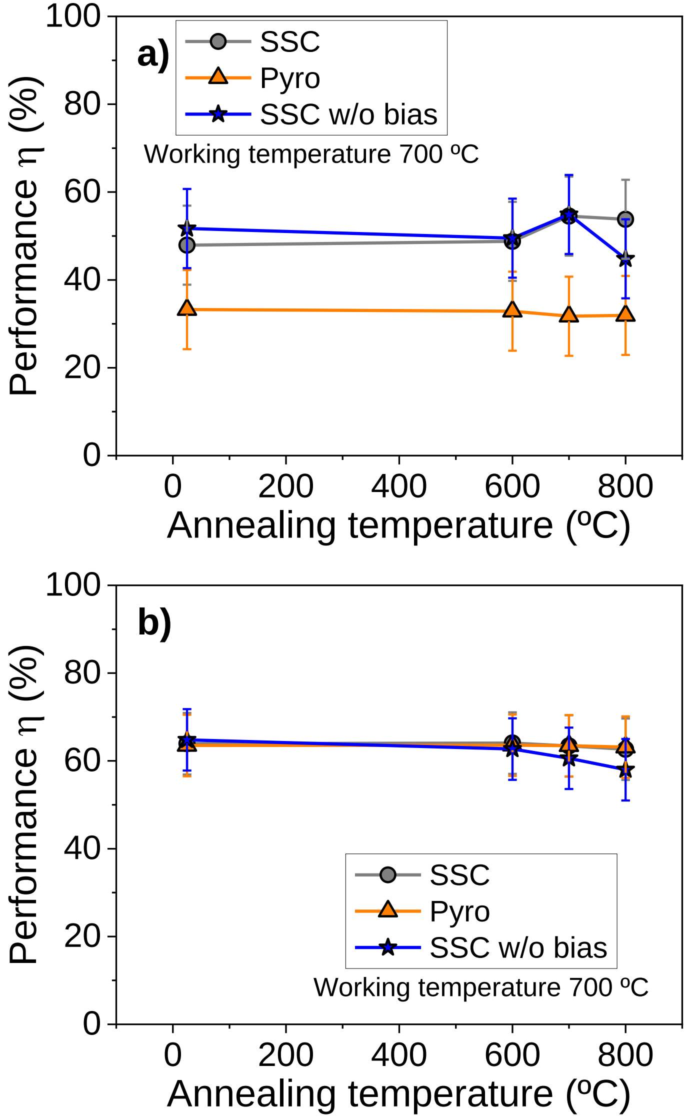

Fig. S4: Evolution of the solar performances η of the annealed stacks on both type of substrates (SS316 and INC625) calculated at target working temperatures of 600, 700 and 800 °C at two concentration factors: C = 100 (a,b,c) and C = 1000 (d,e,f).

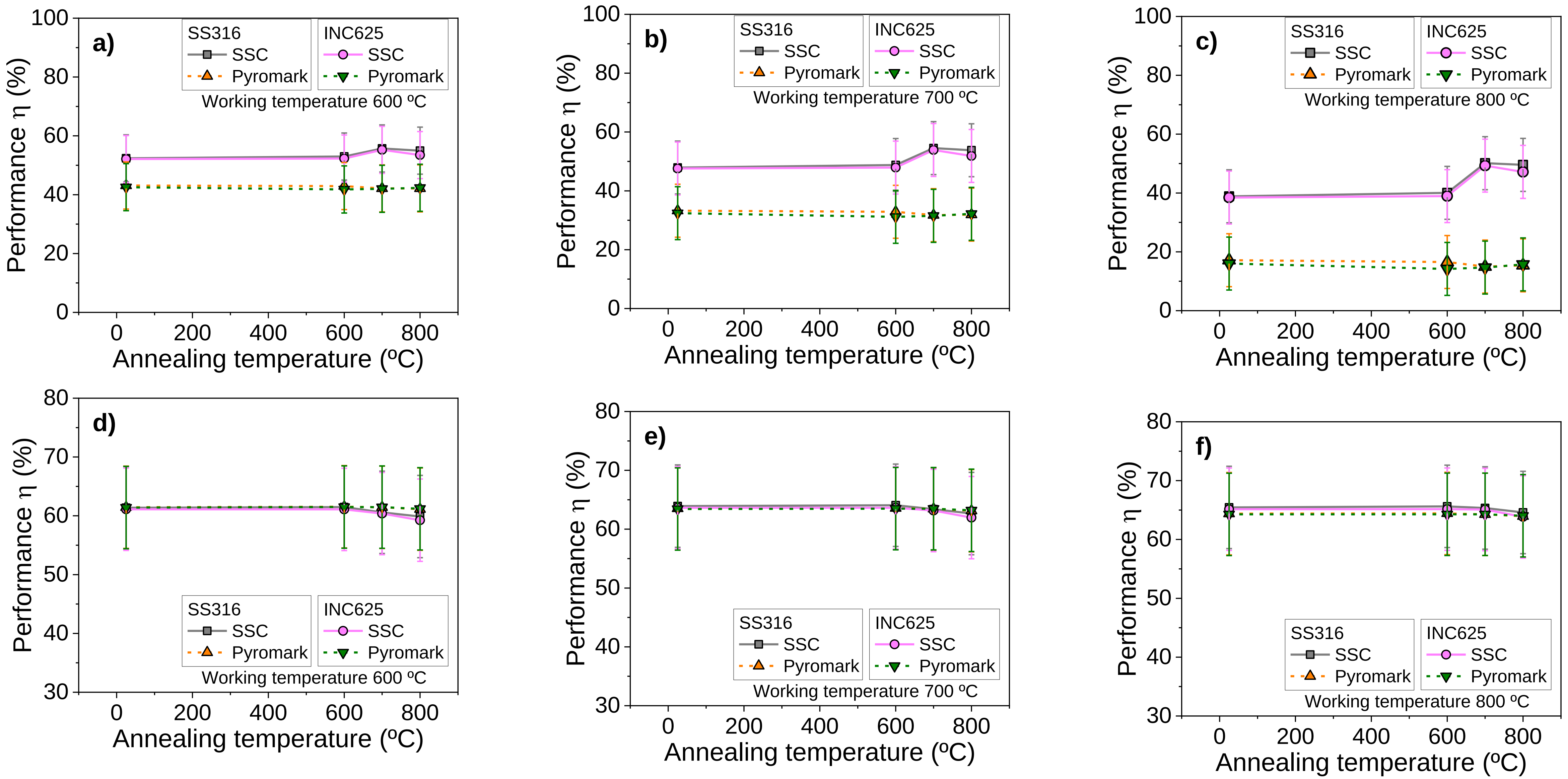

Fig. S5: Calculated solar performances η of the solar selective stack deposited on both substrates (SS316 and INC625) after annealing in air at 600 °C during 200 h at target working temperatures of 600, 700 and 800 °C for a concentration factors: C = 100 (a) and C = 1000 (b).

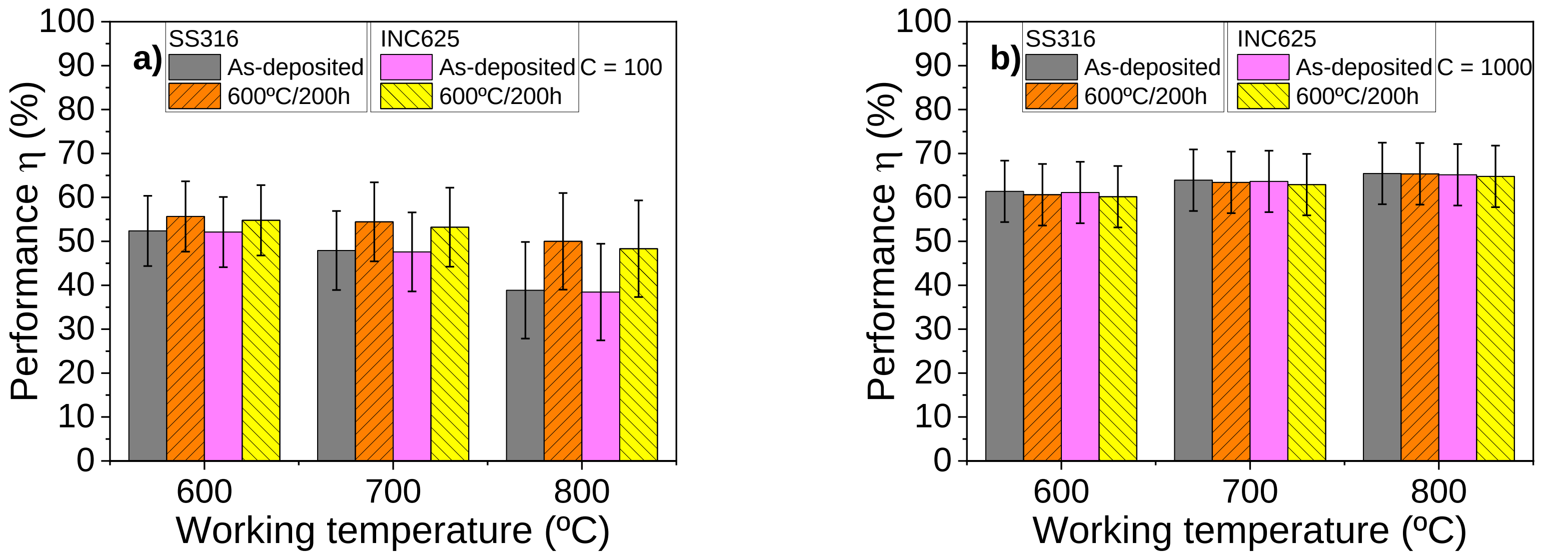

Supplement: Supplementary file 1 — ae3c02310_si_001.pdf [file ae3c02310_si_001.pdf]
